# Inter-individual variability in neural response to low doses of LSD

**DOI:** 10.1038/s41398-024-03013-8

**Published:** 2024-07-15

**Authors:** Nadia R. P. W. Hutten, Conny W. E. M. Quaedflieg, Natasha L. Mason, Eef L. Theunissen, Matthias E. Liechti, Urs Duthaler, Kim P. C. Kuypers, Valerie Bonnelle, Amanda Feilding, Johannes G. Ramaekers

**Affiliations:** 1 Department of Neuropsychology & Psychopharmacology, Faculty of Psychology & Neuroscience, Maastricht University, Maastricht, the Netherlands; 2 Division of Clinical Pharmacology and Toxicology, Department of Biomedicine and Clinical Research, University Hospital Basel, University of Basel, Basel, Switzerland; 3 The Beckley Foundation, Beckley Park, Oxford, UK

**Keywords:** Neuroscience, Psychology

## Abstract

The repeated use of small doses of psychedelics (also referred to as “microdosing”) to facilitate benefits in mental health, cognition, and mood is a trending practice. Placebo-controlled studies however have largely failed to demonstrate strong benefits, possibly because of large inter-individual response variability. The current study tested the hypothesis that effects of low doses of LSD on arousal, attention and memory depend on an individual’s cognitive state at baseline. Healthy participants (N = 53) were randomly assigned to receive repeated doses of LSD (15 mcg) or placebo on 4 occasions divided over 2 weeks. Each treatment condition also consisted of a baseline and a 1-week follow-up visit. Neurophysiological measures of arousal (resting state EEG), pre-attentive processing (auditory oddball task), and perceptual learning and memory (visual long-term potentiation (LTP) paradigm) were assessed at baseline, dosing session 1 and 4, and follow-up. LSD produced stimulatory effects as reflected by a reduction in resting state EEG delta, theta, and alpha power, and enhanced pre-attentive processing during the acute dosing sessions. LSD also blunted the induction of LTP on dosing session 4. Stimulatory effects of LSD were strongest in individuals with low arousal and attention at baseline, while inhibitory effects were strongest in high memory performers at baseline. Decrements in delta EEG power and enhanced pre-attentive processing in the LSD treatment condition were still present during the 1-week follow-up. The current study demonstrates across three cognitive domains, that acute responses to low doses of LSD depend on the baseline state and provides some support for LSD induced neuroadaptations that sustain beyond treatment.

## Introduction

Self-reported use of low doses of psychedelics (also referred to as “microdosing”) like lysergic acid diethylamide (LSD) or psilocybin to enhance mental health, cognition, and mood, has gained increased scientific attention over the last years [1]. Only a few studies however have been conducted to validly assess the benefits of such practices in placebo-controlled designs. Some of these studies have indeed reported small improvements in attention, emotional processing, and mood after administration of a low dose of a psychedelic in healthy volunteers [2–5], but other studies have failed to replicate such findings [6, 7] or have noted that performance enhancement appears to be most prominent in participants breaking blind and therefore might be driven by expectancy [8–10].

It has been shown that a large variation exists in the cognitive and subjective response of individuals to a low dose of a psychedelic and that some individuals may show improvement of cognition and mood whereas others do not [2]. Inter-individual response variations may arise from a number of contributory factors such as varying dose strength, drug concentration in blood [2, 7], genetic differences in drug metabolism and receptor interactions [2, 11], personality [12] and setting [13, 14]. Additionally, an individual response to a low dose of a psychedelic might also depend on arousal levels at baseline [15]. Previous work on stimulant drugs has revealed that performance-enhancing effects are largest in individuals with low baseline arousal states, and virtually absent in highly aroused individuals [16–19].

Arousal states ranging from full alertness to sleep can objectively be measured with resting state electroencephalography (EEG) which provides a general measure of brain activity [20, 21]. Decrements in power in low-frequency bands (i.e., delta, 1–4 Hz; theta, 4–8 Hz; alpha, 8–13 Hz), as well as increments in high-frequency bands (i.e., beta, 13–30; gamma, 30–45 Hz), have been associated with higher levels of wakefulness [20, 22, 23]. EEG paradigms can also be employed to assess variations in event related potentials (ERPs) reflecting attentional and memory processes that might be affected by low doses of LSD. For instance, mismatch negativity (MMN), the primary ERP outcome measure of the roving oddball paradigm, provides a sensory marker of pre-attentive processing and attentional switching during information processing tasks [24, 25]. The visual Long Term Potentiation (LTP) paradigm, measures increased neural activation to visual stimuli after repeated stimulation and is recognized as a core neuronal process underlying neuroplasticity, learning and memory potentiation [26].

The current study aimed to examine inter-individual variation to low doses of LSD on a broad range of cognitive domains as assessed with neurophysiological measures of arousal (resting state EEG), pre-attentive processing (auditory oddball task), and perceptual learning and memory (visual LTP). Based on previous studies showing that a low dose of LSD (13 and 26 mcg tartrate) reduced broadband oscillatory power [27] and that psilocybin mushrooms (0.5 g, dried) reduced EEG theta band power [10], we expected that repeated doses of LSD would reduce resting state EEG power in the low-frequency bands to reflect an overall increase in arousal. In addition, we expected that stimulatory effects of LSD would be stronger in individuals with high EEG power (i.e., low arousal) in low-frequency bands at baseline. In regard to ERP measures, previous studies have shown increased as well as blunted responses after low doses of LSD (13 and 26 mcg tartrate) during reward feedback processing [28] and during an emotional oddball task [27] respectively. In the present study, we did not have a specific expectation on the directionality of the effect of low doses of LSD on ERP parameters in the auditory oddball and visual LTP paradigms. However, we did expect that the magnitude of the LSD effect on ERP parameters would be highest in individuals with the lowest or highest baseline (task) performance, depending on the directionality of the LSD effect.

## Materials and methods

### Participants

The study was conducted according to the code of ethics on human experimentation established in the declaration of Helsinki and subsequent amendments [29]. It was approved by the Medical Ethics Committee of the Academic Hospital of Maastricht and Maastricht University and registered in the Netherlands Trial Register (number: NTR 8736 https://www.onderzoekmetmensen.nl/). Participants received monetary compensation per hour invested.

Healthy participants were recruited to participate in this study through advertisements in university buildings in Maastricht, via social media, local newspapers, and by word of mouth. Before inclusion, participants underwent a medical screening. General health was checked, and blood and urine samples were taken for standard blood chemistry, hematology, and urinalysis. For details on the inclusion and exclusion criteria see Supplementary Materials.

### Study design

The study was conducted according to a double-blind, placebo-controlled, between-subjects design in which the participants received either 4 times a placebo or 4 times a low dose of LSD base (15 mcg) orally divided over 2 weeks. Baseline and follow-up assessments were taken 1 week before and 1 week after the LSD dosing period. EEG measures were taken at baseline, after the first and fourth dosing sessions, and at follow-up. Participants were randomly allocated to one of the two treatment conditions. LSD doses were dissolved in 0.6 mL ethanol (96% Vol). The placebo solution consisted of 0.6 mL of ethanol only [30]. A permit for obtaining, storing, and administering LSD was obtained from the Dutch Drug Enforcement Administration.

### Procedures

**Fig. 1:**
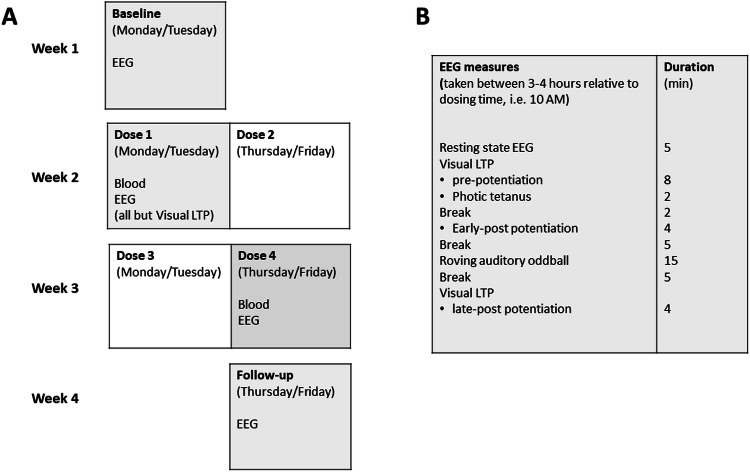
Study design. **A** Schematic presentation of the 4-week treatment schedule consisting of baseline, 4 dosing days (LSD or placebo) and a 1 week follow-up session. EEG data was collected on 4 days (shaded in gray). **B** Time schedule of EEG measures. LTP long-term potentiation.

During the 2 weeks prior to study entrance, a training session of about 4 h took place in order to familiarize participants with all tasks and task procedures. The precepts to which the participant adhered are presented in the Supplementary Materials. Participants attended four 6-hour sessions (baseline, dose 1, dose 4, and follow-up) during which EEG measures were taken between 3–4 h relative to time of dosing, and two 1.5-hour sessions during which participants received doses 2 and 3 (Fig. 1). At arrival, participants were screened for the presence of alcohol in their breath, drugs of abuse in their urine (THC/ opiates/ cocaine/ amphetamines/ methamphetamines), and women were tested for pregnancy. Treatment administration occurred only when screening results were negative. Treatments were administered at 10 AM. Data presented here are part of a larger study on the impact of repeated LSD dosing on psychometric, cognitive and autonomic function. These data were repeatedly collected during the 6 h time window after dosing. Only EEG will be presented here.

### EEG measures

Resting state EEG data were recorded continuously while participants rested with their eyes open (EO) staring at a fixation cross in the center of the computer screen for 2.5 min, followed by eyes closed (EC) for 2.5 min. Participants were instructed not to think about anything in particular, to stay awake, and minimize excessive eye blinking and movement of the body. The primary outcome measure was the oscillatory power of five frequency bands (delta, 1–4 Hz; theta, 4–8 Hz; alpha, 8–13 Hz; beta, 13–30 HZ; and gamma, 30–45 Hz) across scalp electrodes related to the default mode network (i.e., F3, Fz, F4, C3, Cz, C4, P3, Pz, P4, and POz) [31].

The roving auditory oddball task was used to probe the mismatch negativity (MMN), P3a, and repetition suppression in response to unattended auditory stimuli [32]. The task was run in MATLAB using the Cogent toolbox. The stimuli consisted of trains of 6 to 10 identical sinusoidal tones. The first tone of each train typically produces the classic oddball response or MMN. This tone is referred to as the deviant tone, while the 5th tone typically produces the standard response and is therefore referred to as such [33]. Pseudo-randomisation of train length produced 208 deviant presentations that were repeated 5 times; in addition the tone was repeated 1–4 times randomly but equally often, meaning all trains had 6–10 tone repetitions. The tones were 70 ms in length with a 5 ms rise and fall time, and a 500 ms inter-stimulus interval. To produce the deviant presentation, the tone frequency varied between 500 and 800 Hz in random steps of integer multiples of 50 Hz. Tones were presented binaurally at a constant volume that was adjusted for individual participants so that it was clear and comfortable. Participants were instructed to ignore the auditory tones and to focus on the visual distractor task (for more information see Supplementary Materials). Latency and amplitude of the MMN and P3a were calculated for the following electrodes: F3, Fz, F4, C3, Cz, C4, P3, Pz, P4, and POz [34].

Visual long-term potentiation (LTP) was measured with a paradigm used in previous studies [35–37]. Stimuli for this task were vertical and horizontal sine gratings with a spatial frequency of 1 cycle per degree. The sine gratings were presented at full contrast on a gray background, subtending 8° of visual angle. Participants were seated with their eyes 57 cm from the center of the screen and were instructed to passively fixate on a central red dot. The task comprised of four conditions. During the first condition (pre-potentiation), both stimuli were presented 120 times in random order at 1 Hz for 34.8 ms. The interstimulus interval was varied using 5 intervals from 897 to 1036 ms that occurred randomly but equally often. This condition established the baseline ERP amplitude for subsequent comparison with post-potentiation conditions and had a duration of 8 min. The second condition was a 2-minute photic tetanus or high-frequency stimulation comprising 1000 presentations of either the horizontal or vertical stimulus to test input specificity (counterbalanced between participants) for 34.8 ms with a temporal frequency of approximately 9 Hz. The interstimulus interval was either 62.6 or 90.4 ms occurring at random but equally often. The third condition (early post-potentiation) followed after a 2-minute break, allowing retinal after-images from the photic tetanus to dissipate. The fourth condition (late post-potentiation) followed ~30 min after the photic tetanus. The two post-conditions were identical to the pre-potentiation condition, and lasted 4 min each. Amplitude and latency were calculated for the following electrodes: P7, P3, Pz, P4, P8 and POz [38, 39]. Details on EEG data acquisition and analysis are provided in the Supplementary Materials.

### LSD concentration and treatment guess

Blood samples were taken 2 h after dose 1 and dose 4. The blood was centrifuged, and pipetted plasma was frozen at −20 °C until analysis. LSD and O-H-LSD concentrations were determined using ultra high-performance liquid chromatography-tandem mass spectrometry (UHPLC–MS/MS) as previously described [40]. Subjects were asked at the end of dosing days 1 and 4 to guess whether they were randomized to the LSD or the placebo group.

### Statistical analyses

Sample size was based on a G-power analysis for F-tests in a mixed between/within repeated measures design for detection of changes with a low effect size (i.e.0.25), 80% power and alpha=0.05. Data were analyzed by means of the statistical package IBM SPSS Statistics (version 27). All EEG data were analyzed with linear mixed models (LMMs) with a restricted maximum likelihood method (REML). An unstructured covariance structure was used. Missing data were handled using listwise deletion.

LMMs were conducted on baseline EEG with Treatment (2 levels), Electrode (levels depending on the EEG task), and Treatment by Electrode as fixed effects. For the visual LTP task, a factor Time (3 levels of potentiation: pre, early-post, late-post) was added. In the absence of baseline differences, subsequent LMMs were conducted with model parameters Treatment (2 levels), Test day (3 levels: dose 1, dose 4, and follow-up), Electrode (levels varied per EEG task), Treatment by Test day, Treatment by Electrode, and Test day by Electrode as fixed effects. In case of a significant Treatment by Test day interaction, Bonferroni-corrected pairwise comparisons were performed between treatments on each test day. For further details on additional model parameters for the auditory roving oddball task and the visual LTP, see Supplementary Materials.

Correlational analyses (Bonferroni corrected) were performed between EEG/ERP measures at baseline (averaged across electrodes) and treatment-induced changes in EEG/ERP parameters (means of sessions 1 and 4 minus baseline averaged over electrodes) to assess the association between inter-individual variation in baseline EEG/ERP and outcome EEG/ERP measures. Pearson correlations were used in normally distributed data and Kendall’s Tau-b in non-normally distributed data. Potential effects of regression to the mean that may confound this procedure are controlled by design as baseline associations are established within the LSD group as well as the placebo control group.

## Results

Two participants dropped out due to Covid restrictions and one participant because of non-adherence to the protocol. Three participants were excluded from all analyses because of missing data caused by a technical malfunction or extreme noise during EEG recordings. Demographic details of the remaining 47 participants are presented in Table 1.

**Table 1:** Demographics of study participants.

	Placebo (N = 24)	LSD (N = 23)
Sex (male/female)	12 / 12	12 / 11
Mean age ± SD (min-max)	36.3 ± 18.2 (18–64)	35.9 ± 14.4 (21–63)
*Educational level*^a^		
Higher education	16	14
Vocational/secondary education	8	9
*Prior experience with psychedelics*^a^		
2-CB	1	0
Ayahuasca	2	1
DMT	1	0
Ketamine	1	3
LSD	5	6
Psilocybin	12	8
Khat	0	1
XTC (MDMA)	5	8
No prior experience with psychedelics	11	14

^a^Data are number of participants

### Resting-state EEG

LMMs revealed no significant baseline differences between treatment conditions for all resting state EEG power frequencies for both EO and EC conditions across all electrodes (See Table S1).

**Fig. 2:**
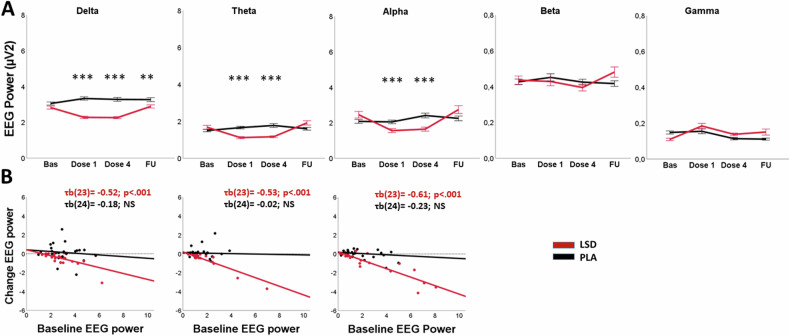
Resting state EEG. **A** Mean (SE) resting state EEG power for the LSD and placebo conditions per test day. **B** Scatterplots of mean change in resting state delta, theta and alpha EEG power (the mean over the two dosing days minus baseline) for the LSD and placebo condition as a function of baseline resting state EEG power. Bas=baseline, FU=Follow-up. Eyes open condition; ***p < 0.001; **p < 0.01.

LMMs revealed significant main effects for Treatment for delta (all F > 52.55; all p < 0.001) and theta (all F > 6.16; p < 0.01) power in the EO and EC condition. Significant main effects of Treatment were also found in gamma (EO, F_1,453,37_ = 5.75; p = 0.017) and alpha (EC, F_1,450.95_ = 6.58 p = 0.011) power. Main Treatment by Test day interaction effects were found for all resting state frequencies for both EO and EC conditions (all F > 7.77; all *p* < 0.001), except for beta (EC) and gamma (EO) (Table S2). Pairwise comparisons revealed that the LSD group had lower delta (EO&EC; all p < 0.001), theta (EO&EC; all p < 0.001) and alpha (EO; all p < .001) power during dosing sessions 1 and 4, and lower delta (EO, p = 0.002; EC, p = 0.008) on the follow-up test day compared to placebo (EO: Fig. 2A; EC: Fig. S1a). Overall, alpha power (EC) was higher on each test day for the LSD group compared to the placebo group, but achieved significance only on dosing session 1 (p = 0.011) and follow-up (p = 0.003). Visual inspection (Fig. S1) however demonstrates that LSD alpha power in the EC condition did not increase relative to LSD baseline but was higher relative to the placebo group across all testing days, suggesting a group difference (despite a non-significant difference at baseline) rather than an actual treatment effect. Gamma power (EC) was higher during dosing session 1 in the LSD group compared to the placebo group (p = 0.015). Gamma power (EO) did not differ between LSD and placebo on separate test days.

In the LSD condition, Bonferroni corrected correlations revealed that baseline delta (EO, τb(23) = −0.52, p = 0.000 & EC, τb(23) = −0.52, p = 0.007), theta (EO, τb(23) = −0.53, p = 0.000 & EC τb(23) = −0.61, p = 0.000) and alpha (EO; τb(23) = −0.61; p = 0.000) power (averaged over electrodes) were negatively associated with the treatment-induced change from baseline in oscillatory power (EO: Fig. 2B; EC Fig. S1b), suggesting that higher baseline oscillatory power was associated with a larger decrease in the respective oscillatory power following acute doses of LSD. The change in alpha (EC), beta (EO & EC) and gamma (EO, EC) power did not significantly correlate with baseline power.

### Roving auditory oddball task

LMMs revealed that in both treatment conditions and on all test days, there was a significant difference between the deviant (tone 5) and standard stimulus (tone 1) for both the MMN at N170 (*F*_1,747.55_ = 849.62, *p* < 0.01) and P3a (*F*_1,763.22_ = 924.38, *p* < 0.01) amplitudes across all electrodes, indicating that the oddball paradigm was successfully implemented (see also Supplementary Materials). At baseline, LMMs on the difference waves (tone 1 - tone 5) of the MMN and the P3a, revealed a significant Treatment by Electrode interaction effect on the MMN latency (*F*_8,45_ = 4.08, *p* < 0.01), indicating that the MMN appeared significantly earlier in the LSD condition compared to the placebo condition. This however was limited to the F3 electrode. There were no further significant main effects of Treatment or Treatment by Electrode on the MMN and the P3a voltage and latency at baseline (Table S3).

**Fig. 3:**
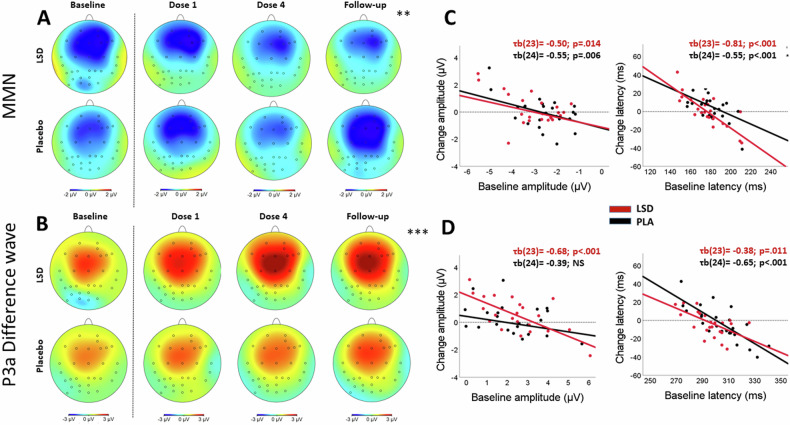
Roving auditory oddball task. **A** Topographies of the MMN amplitude at 120–250 ms after stimulus presentation. **=p <0.01 at follow-up. **B** Topographies of the P3a difference wave amplitude after 250–350 ms. ***p = <0.001 across Dose 1, Dose 4 and follow-up. **C** Scatterplots of the change in amplitude and latency of the MMN for the LSD and placebo condition as a function of baseline amplitude and latency. **D** Scatterplots of the change in difference wave amplitude and latency of the P3a for the LSD and placebo condition as a function of baseline amplitude and latency.

LMMs revealed a significant Treatment by Test day interaction on the amplitude of the MMN difference wave across all electrodes (F_2,381.67_ = 7.05; p = 0.001; Table S4). Bonferroni-corrected pairwise comparisons revealed that the amplitude of the MMN was significantly less negative in the LSD condition compared to placebo on the follow-up test day (*p* = 0.005) (Fig. 3A). There was a main effect of Treatment for amplitude of the P3a, (F_1,273.75_ = 21.63; *p* < 0.001) that was more positive in the LSD group compared to the placebo group (Fig. 3B). For the latency, there was a main effect of Treatment for both the MMN (F_1,407.58_ = 35.9; *p* < 0.001) and P3a (F_1,401.91_ = 26.81; *p* < 0.001) (Table S5). The MMN and the P3a appeared earlier in the LSD condition. There were no significant Treatment by Electrode interactions for latencies and amplitude of the MMN and P3a. The estimated means of the MMN and P3a voltage and latency per treatment condition per test day, across all electrodes, are presented in Table S5.

For amplitude and latency (averaged across electrodes), Bonferroni corrected correlations between baseline MMN and the P3a and treatment-induced change across the acute dosing sessions are shown in Fig. 2C, D. Treatment-induced changes in latency were negatively correlated to baseline latency of the MNN and the P3a component (all p < 0.011). Likewise, treatment-induced change in amplitude was negatively correlated to the baseline amplitude of the P3a component in the LSD condition (τb(23) = −0.68; p < 0.001), and to the baseline amplitude of MMN in both treatment conditions (LSD, τb(23) = −0.50; p = 0.014; placebo, τb(24) = −0.55; p = 0.006)

Overall, there was no repetition suppression effect present on any test day in the placebo condition, indicating that on all test days, the amplitude of the N170 and the P3a habituated after one repetition. As for the LSD condition, on baseline and dose 1, habituation of the N170 amplitude was present after two repetitions. During dose 4 and the follow-up test day, the P3a amplitude was habituated after two repetitions in the LSD group. Details of the results regarding the analyses of the repetition suppression effect are presented in the Supplementary Materials Table S6 and Fig. S2.

### Visual long-term potentiation

**Fig. 4:**
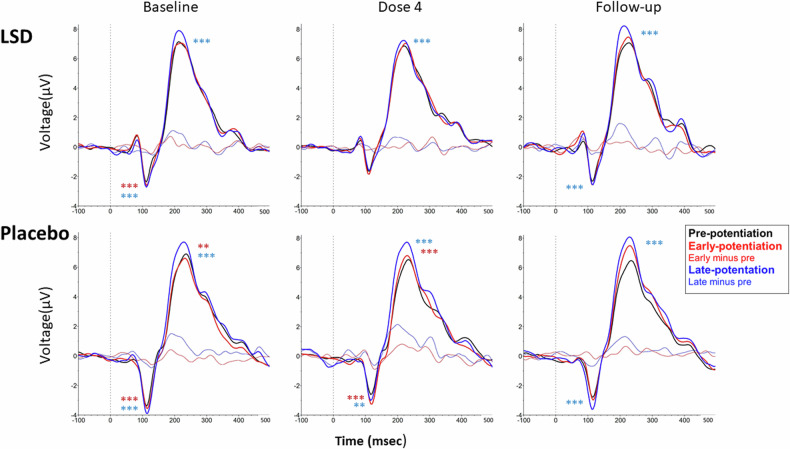
Visual LTP. ERPs (averaged across tetanized and non-tetanized) of the visual LTP task per treatment condition (LSD and placebo), on baseline, dose 4 and follow-up test day depicted at a single electrode (POz). The blue asterisks indicates a significant difference between late and pre-potentiation, the red asterisks indicates a significant difference between early and pre-potentiation across all electrodes. ***p < 0.001; **p < 0.01.

LMMs were used to investigate the effect of Time (pre-potentiation, early-post, and late-post potentiation) and LTP Input specificity (tetanized vs non-tetanized) to inform the parameters of the subsequent analysis. Overall, for both the N1 and P200 amplitudes, there was no effect of Input specificity, only an effect of Time (all F > 3.58; all *p* < 0.01), meaning that LTP occurred regardless of whether the stimulus was tetanized or non-tetanized (Supplementary Materials). Therefore, all further analyses were conducted with the averages across the tetanized and non-tetanized stimuli. A graphical representation of the LTP inductions at POz at every test day in each treatment condition is given in Fig. 4.

At baseline, LMMs revealed no differences in the N1 and the P200 amplitudes and latencies and the amplitudes and latencies of the difference waves (late-potentiation minus pre-potentiation) between treatment conditions (Supplementary Materials). The interaction between Treatment and Electrode also did not reveal any difference. These results indicate that at baseline for both the N1 and P200 there was a significant LTP induction across electrodes and in both treatment groups.

For the N1 amplitude, a significant Treatment by Test day by Time interaction (*F*_2,1046.70_ = 4.07; *p* < .017) was found. Both treatment conditions produced similar amplitude modulations during the follow-up, but their modulation on dosing session 4 differed (F_314,278_ = 9.97 *p* < 0.001). Bonferroni corrected pairwise comparisons showed that at dosing session 4, the N1 amplitude during early and late potentiation was significantly more negative as compared to pre-potentiation in the placebo condition (all p < 0.013) but not in the LSD condition (Fig. 4). The amplitude of the N1 difference waves revealed a significant Treatment x Test day interaction (*F*_1,1095.14_ = 4.88; *p* = 0.027), but subsequent contrasts did not reveal a difference between treatment conditions on separate test days.

**Fig. 5:**
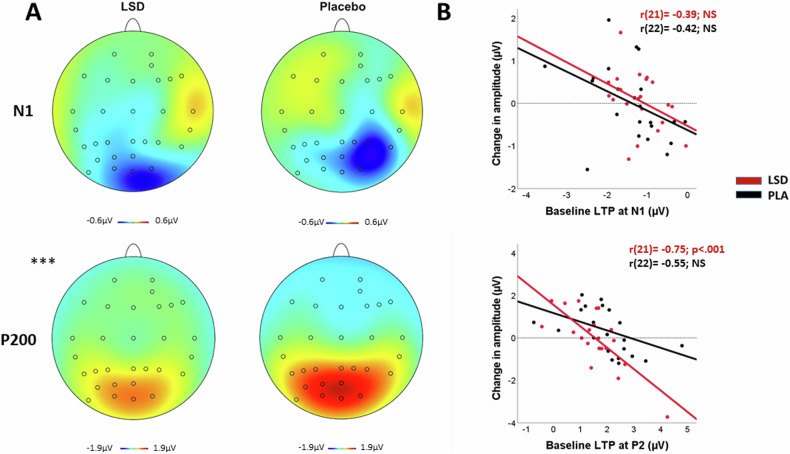
Visual LTP. **A** Topographies of the difference waves for N1 (100–190 ms) and P200 (190–280 ms) comparing late-potentiation with pre-potentiation (LTP), per treatment (LSD and placebo) at dosing session 4. **B** Scatterplots of the treatment induced change in LTP at the N1 and P200 amplitudes for the LSD and placebo condition, as a function of LTP at baseline. ***= p ≤ 0.001.

The P200 amplitude of the difference waves showed a Treatment by Test day interaction (*F*_1,1084.31_ = 27.36; *p* < 0.001). Bonferroni corrected contrasts revealed that the P200 amplitude was lower on dosing session 4 in the LSD condition (*p* = .001) as compared to placebo (Fig. 5A), but similar during the follow-up of both conditions.

None of the LTP parameters revealed a Treatment by Electrode interaction. Details of the LMMs for both the N1 and P200 amplitudes and latencies across both treatment conditions are presented in the Supplementary Materials.

Correlation analyses between baseline difference waves of the P200 and the N1(late-potentiation minus pre-potentiation, averaged across electrodes) with treatment-induced change from baseline in difference wave at dosing session 4 are shown in Fig. 5B. There was a significant correlation in the LSD condition at the P200 amplitude (*r*(21) = −0.75, *p* < 0.001), indicating that those who showed a larger LTP induction at baseline, showed a larger reduction in LTP while under the influence of LSD on dose session 4. There were no significant correlations at the N1 amplitudes.

### Treatment blinding and LSD concentrations

Treatment guesses were reported at the end of dosing session 1 and 4. Consistent guesses on dosing sessions 1 and 4 were classified as correct or incorrect. Some participants guessed differently on dosing sessions 1 and 4, indicating that they were unsure about their treatment allocation. Such guesses were classified as inconsistent. In the placebo group, the frequency distribution of correct (57.1%) and incorrect/inconsistent treatment guesses (42.9%) did not significantly differ (χ2 = 1; p = 0.32). In the LSD group, the frequency distribution of correct (59.6%) and incorrect/inconsistent treatment guesses (40.4%) also did not differ (χ2 = 1.9; p = 0.17).

Mean (SE) concentrations of LSD 2 h after dose 1 and dose 4 were 302 (105) pg/mL and 326 (117) pg/mL, respectively. Mean (SE) concentrations of O-H-LSD after Dose 1 and Dose 4 were 17 (7.2) pg/ml and 17 (4.9) pg/mL, respectively.

## Discussion

The present study aimed to investigate the neural underpinnings of inter-individual variation in cognitive responses to low doses of LSD as assessed with neurophysiological measures. Overall, LSD reduced resting state EEG delta, theta, and alpha power during the acute dosing sessions compared to placebo. Delta power remained lower in the LSD group during follow-up. During dosing sessions, individuals with high EEG (delta, theta, alpha) power at baseline showed larger decrements in EEG power under LSD. On dosing sessions and during follow-up, the latencies of the MMN and the P3a of the auditory oddball task appeared earlier in the LSD condition, and the amplitude of the P3a was more positive compared to the placebo. The MMN amplitude was also higher after LSD but only during follow-up. Across dosing sessions, treatment-induced changes in these parameters were negatively correlated with their baseline equivalent after both LSD and placebo, but most often after LSD. The LTP induction at the P200 was significantly lower in the LSD condition compared to the placebo condition during the fourth dosing session. Participants that showed a large LTP P200 at baseline showed a larger inhibition of LTP induction in the LSD condition. Plasma concentrations of LSD 2 h after administration, which is shortly after the time to reach maximal concentrations, were in the expected range compared to studies using 10 or 20 mcg of LSD [41].

The reduction in resting state EEG power in the low-frequency bands (1–13 Hz) following low doses of LSD is in line with previous studies using low doses of LSD (13 and 26 mcg tartrate) [27] and dried psilocybin mushrooms (0.5 g) [10] as well as with studies using full doses of psychedelics [42–45]. Decrements in resting state EEG power in low-frequency bands have repeatedly been associated with higher levels of arousal and wakefulness, for instance after caffeine intake [46–48] and stimulants such as dexamphetamine [49]. In the present study, LSD-induced decrements in EEG power were negatively correlated with EEG power at baseline, indicating that the stimulant effects of LSD were stronger in individuals with low arousal levels (i.e., having high power in low frequency bands) at baseline. Pharmacological mechanism underlying the stimulatory action on arousal of low doses of LSD may involve dopaminergic receptor modulation [50]. Preclinical studies have shown that LSD may affect frontostriatal dopamine, via direct or indirect stimulation of striatal dopamine receptors [51, 52]. Dopaminergic effects of low doses LSD have also been speculated to underlie increased reward related brain activity in humans [28]. Alternatively, psychedelics are also known to release cortisol in humans [53–55] which may induce a stress mimicking effect, that latter of which has been associated with decrements in EEG power of low-frequency bands [56]. Though both explanations may account for the stimulatory effects of LSD observed after an acute dose, they are unlikely to account for sustained levels of arousal (i.e. decrements in delta power) hat were observed at the 1 week follow-up. The latter might be more related to persisting changes in neuroplasticity [57] or the immune profile [55] that have been reported after single doses of psychedelics.On dosing sessions and at follow-up, the MMN and the P3a latencies appeared earlier in the LSD condition, while the amplitude of the P3a was more positive compared to placebo. The MMN amplitude was also higher in the LSD condition but only during follow-up. These findings suggest that novelty detection and preattentive processing were improved in the LSD treatment condition. Stimulatory effects of LSD were most pronounced in individuals with poorer preattentive processing at baseline as expressed by a significant correlation between baseline latencies/amplitude of MMN and P3a and LSD induced change. It is noteworthy that a similar correlation was found for MMN and P3a latencies in the placebo group, suggesting that such associations might not be solely treatment related and could also reflect additional underlying factors such as practice. However, the association between baseline P3a amplitude and treatment induced change was only significant in the LSD group, supporting the notion that LSD effects on neural performance in the auditory oddball task varied with baseline. These findings are in line with resting state EEG data showing baseline dependent stimulatory effects under LSD. The findings are in contrast however with a previous study showing that low doses of LSD (13 and 26 μg tartrate) decreased ERP amplitudes and increased latency in an emotional faces oddball task [27]. The latter paradigm, however, required additional cognitive, affective and perceptual processing associated with facial recognition, in comparison to the current auditory paradigm. Stimulatory effects of low doses of LSD on MMN are also in contrast with previous studies showing a blunted MMN following moderate to high doses of DMT, LSD, and (es)ketamine [44, 58–61] or the absence of an effect of psilocybin on MMN [58, 59, 62]. At higher doses, psychedelics have been suggested to increase bottom-up processing of sensory information [63, 64] and relax top-down control [65] in healthy volunteers that may lead to a sensory overload and a subsequent breakdown of sensory integration as reflected by impaired MMN [64]. In depressed patients, on the other hand, treatment with ketamine was shown to improve MMN presumably by increasing top-down prediction error sensitivity [66]. The impact of psychedelics on measures of pre-attentive processing may therefore vary with dose and individual information processing capacities, such as the predictive coding of incoming sensory input [67], and differ between oddball paradigms that may tap into sensory and cognitive processes to varying degrees. The present data adds that low doses of LSD can subtly accelerate and improve the processing of auditory sensory information, at least in healthy volunteers.

Overall, LSD reduced LTP P200 during the 4th dosing session as compared to placebo. This reduction in LTP was larger in participants who showed a larger LTP induction at baseline, suggesting that inhibitory effects of LSD were strongest in participants with higher levels of perceptual learning and memory at baseline. Inhibitory effects of low doses of LSD on memory processes do not come as a big surprise, as moderate to high doses of LSD and other psychedelics such as psilocybin have been demonstrated to produce memory impairment acutely [68–70]. Inhibitory effects may result from a change in balance of glutamateric and GABAergic input to the thalamocortical circuitry that underlies LTP [71]. Psychedelics have been shown to acutely alter excitatory glutamate concentration in a regional dependent manner, with increments observed in the medial prefrontal cortex and reductions in the hippocampus [72]. Acute impairing effects of LSD on memory however are transient, and some evidence even suggests that memory may improve subacutely [73] through stimulation of neuroplasticity [74]. The LTP paradigm did not provide any supporting evidence for increased synaptic connectivity in neural sensory circuits however, as we did not observe any improvement in LTP induction under LSD. Yet, a previous study has shown that low doses of LSD may indeed increase neuroplasticity within 2–6 h of administration as shown by acute increments in BDNF [2]. A higher dose of LSD might be needed to also increase LTP induction, as previously shown with a dissociative dose of ketamine in a depressed patient sample [66].

The present dataset reconfirms that low doses of LSD can reduce oscillatory EEG power and modulate event-related potentials related to preattentive processing and perceptual learning, but also adds two major findings. First, the data suggests that neural effects produced at a low dose of LSD differ between individuals and relate to their cognitive state at baseline. Stimulatory effects of LSD were most pronounced in individuals displaying low arousal (resting state EEG) and low pre-attentive performance (Roving auditory oddball task) at baseline, while the impairing effects of LSD in LTP were stronger in individuals that scored high on perceptual learning and memory. In other words, the effects of a low dose of LSD were maximal in individuals with the largest capacity for performance improvement or impairment, depending on the task at hand. Secondly, some of the neural effects that were recorded in the LSD condition (i.e., reduced delta power during resting state and increments in MNN and P3a amplitude during the oddball paradigm) pertained over time and were still noticeable during follow-up, 1 week after the fourth dose. This suggests that the impact of repeated administration of low doses of LSD can pertain beyond the acute effects that are observed on dosing days, at least at the neural level. The presence of prolonged neural effects in the LSD group seems supportive of the notion that repeated administration of low doses may stimulate long-lasting neuroplastic changes in the brain [74, 75]. Whether such neural changes would also translate into subjective and behavioral changes is currently unknown and may depend on the frequency and duration of the dosing scheme.

Neurophysiological effects of low doses of LSD as shown in the present study may also offer vistas for future medical indications such as Attention Deficit Hyperactivity Disorder (ADHD) and Obsessive Compulsive Disorder (OCD) that are characterized by increased EEG power across lower frequency bands and decreased EEG power across higher frequencies [76]. Elevated theta power is a hallmark feature of ADHD [77] that is significantly reduced during successful pharmacological treatment of ADHD symptoms [78]. It is conceivable that a similar reduction in ADHD symptom severity might be achieved with a low dosing regimen of LSD if that results in a (prolonged) reduction of low-frequency EEG power as shown in the present study with healthy volunteers. Retrospective survey data indeed indicate that treatment of ADHD is a major motivation among some psychedelic ‘microdosers’, and that their reported efficacy of low dose psychedelics to reduce ADHD symptoms is equal or even higher as compared to traditional pharmacological treatments [79]. Similarly, a prospective survey among ADHD patients that initiated self-treatment with low doses of psychedelics reported a reduction in ADHD symptoms during a 4-week dosing regimen [80]. Randomized controlled trials in ADHD patients will be needed however to confirm such beneficial findings from observational studies.

Potential limitations of the current study relate to treatment blinding, treatment duration, and treatment population. Treatment unblinding has been identified as a potential bias that might drive subjective changes during psychedelic treatments, even at low doses [8–10]. In the current study however, treatment guesses in the placebo and the LSD group did not exceed chance. This indicates that participants in the placebo and LSD group were well-blinded and not subject to treatment bias. Treatment duration was limited to two weeks in the current study which does not allow for the assessment of cumulative effects of low doses consumed over periods of several weeks or months. Finally, we cannot rule out the possibility that the effects of low doses of LSD would be more prominent in patient populations whose suboptimal baseline capacities may offer more room for improvement.

In sum, the current study confirms that low doses of LSD can increase arousal and pre-attentive processing and can impair perceptual learning and memory as assessed with resting state EEG power and event-related potentials. Across all cognitive domains, LSD induced neurophysiological changes varied between individuals and were strongest in those whose neurophysiological state at baseline offered the most scope for improvement or impairment. Some neurophysiological changes in the LSD treatment condition pertained after the final administration of LSD, suggesting the presence of prolonged neuroadaptations.

### Supplementary information

Inter-individual variability Supplement
