# Supplementary material for: Inter-individual variability in neural response to low doses of LSD
Source: Transl Psychiatry. 2024 Jul 15;14:288. doi: 10.1038/s41398-024-03013-8 (PMC11251148; doi:10.1038/s41398-024-03013-8)
Supplement: Supplementary file 1 — Inter-individual variability Supplement [file 41398_2024_3013_MOESM1_ESM.docx]

**Supplementary Material**

**Inter-individual variability in neural response to low doses of LSD**

Nadia R.P.W. Hutten ^1^, Conny W.E.M. Quaedflieg ^1^, Natasha L. Mason ^1^, Eef L. Theunissen^1^, Matthias E. Liechti^2^, Urs Duthaler^2^, Kim P.C. Kuypers^1^, Valerie Bonnelle^3^, Amanda Feilding^3^, & Johannes G. Ramaekers^1^

**Materials and Methods**

*Participants*

Inclusion criteria were written informed consent; age 18–65 years; proficient knowledge of the English language; good physical and mental health as determined by medical history and medical examination; free from psychotropic medication; BMI between 18 and 28 kg/m2; willing not to drive a traffic vehicle or to operate machines within 24 h after substance administration. Exclusion criteria were a history of drug abuse or addiction as determined by medical history and medical examination; a history of psychiatric and neurological disorders; previous experience with serious side effects to psychedelic drugs (e.g., anxiety or panic attacks); cardiovascular abnormalities; hypertension (diastolic > 90 mmHg; systolic > 160 mmHg); psychotic disorder in first-degree relatives; tobacco smoking (> 20 cigarettes/day); excessive alcohol (> 20 alcoholic consumptions/week); pregnancy or lactation.

*Procedures*

Participants had to refrain from psychedelic substance use three months prior to the study, and from other psychoactive substance use at least one week before entering the study, until completion of all test days. Participants were requested not to consume caffeinated or alcoholic beverages after midnight of the evening before the test days and during the test days. Smokers were asked to refrain from nicotine use 2 hours prior to as well as during the test days. All participants were expected to arrive well-rested at the test facilities in the morning.

*EEG measures and data acquisition*

The electroencephalogram (EEG) was recorded using a BrainAmp amplifier and BrainVision Recorder software (BrainProducts, Germany) from 30 Ag/AgCl electrodes (Fp1, Fp2, AF4, F7, F3, Fz, F4, F6, F8, T7, T8, C3, Cz, C4, TP7, TP8, P7, P5, P3, Pz, P2, P4, P8, PO9, PO7, PO3, POz, O1, Oz, O2) placed in an elastic cap according to the standard international 10-20 system ([Jasper, 1958](https://docs.google.com/document/d/1PTYQj4eS6_iCLTgX6Bo_zLbwO8ZrhO6D/edit#heading=h.26in1rg)). Vertical eye movements were recorded with one electrode above and one electrode below the left eye, and horizontal eye movements were recorded with an electrode at the outer canthi of both eyes. An electrode at AFz served as ground. Data were sampled continuously at 250 Hz, band-pass filtered (0.1–100 Hz), and referenced to the left mastoid (A1) during acquisition. Signals at the right mastoid (A2) and the tip of the nose were also recorded for offline re-referencing. Scalp-electrode impedance was kept below 25 kΩ. When electrode impedances exceeded the threshold, the data was visually inspected to see whether the signal was acceptable. Participants were shown the raw recording signals to demonstrate common artifacts that occur due to body and eye movements. All data were further processed offline using BrainVision Analyzer 2.2 (BrainProducts, Germany).

All data were re-referenced to an average digitally linked mastoids (A1 & A2). In case the signal of A2 contained too much noise, the data was re-referenced to the nose electrode. A notch filter (50Hz) was applied. All data were visually inspected and gross movement artifacts were selected and removed. In case of ECG-contaminated data, independent component analysis (ICA) were used to remove ECG-related artifacts.

Resting-state EEG

Data were band-pass filtered between 0.5-50 Hz, with a cutoff slope of 12 dB and a high cutoff slope of 3 dB. An ocular correction was applied using the Gratton & Coles procedure. Segmentation was performed based on 512 data points to avoid zero padding, resulting in 73 segments with lengths of 2.048 seconds for each condition (EO and EC). Epochs exceeding a ±100 µV at any electrode were excluded from further processing steps. On average, the rejection rate was 1.06% (*SD* = 2.58) of the epochs in the EO condition and 3.33% (*SD* = 4.01) in the EC condition. To calculate the oscillatory power in the delta (1-4 Hz), theta (4-8 Hz), alpha (8-13 Hz), beta (13-30 Hz), and gamma (30-45 Hz), the fast Fourier transform (FFT) was applied with a frequency resolution of 0.488 Hz and a Hanning Window of 10% window length (5% at the beginning and 5% at the end of the segment). Scalp electrodes chosen for the analysis were related to the default mode network; F3, Fz, F4, C3, Cz, C4, P3, Pz, P4, and POz.

Mismatch negativity

For the visual distractor task, a fixation cross was presented in the center of the screen which changed in luminance pseudo-randomly every 2 to 5 seconds. The change in luminance was a subtle change between black and grey, thereby demanding attentional processes from the participant. This visual distractor task was unrelated and not time-locked to the auditory stimuli. To check whether participants were not focusing on the auditory stimuli, participants had to press the spacebar when they noticed the luminance change. The outcome variable was the reaction time (milliseconds).

All data were band-pass filtered between 0.5-30 Hz, with a low cutoff slope of 12 dB and a high cutoff slope of 3 dB. Ocular correction was applied using the Gratton & Coles procedure. Epochs were made for each tone number separately from 100 ms before and 400 ms after tone presentation and were baseline corrected. Epochs exceeding a ±100 µV at any electrode were excluded from averaging. This method of artifact rejection led to a mean rejection of 2.10% (SD = 4.00) of the deviant tone trials and 2.12% (SD = 3.96) of the standard tone trials. Peak detection of the N170 was set between 120-250 ms and the peak of P3a between 250 and 350 ms. Furthermore, difference waves were calculated to examine the magnitude difference between the standard and deviant tone, the mismatch negativity (MMN). Peak detection of these difference waves was extracted as well. Scalp electrodes chosen for the analysis were F3, Fz, F4, C3, Cz, C4, P3, Pz, and P4.

Visual long-term potentiation

All data were band-pass filtered between 0.5-30 Hz, with a low cutoff slope of 12 dB and a high cutoff slope of 3 dB. Epochs were made for each stimulus separately from 100 ms before and 500 ms after stimulus presentation and were baseline corrected. Due to the visual nature of the task, epochs containing eye blinks 100 ms prior and 50 ms after stimulus presentation were removed from further analyses, and the occurring eye blinks after 50 ms were ocular corrected. Epochs exceeding a ±100 µV at any electrode were excluded from averaging. This method of artifact rejection led to a mean rejection of 8.42% (SD = 6.04) of the pre-potentiation trials, 7.78% (SD = 5.83) of the early-potentiation, and 8.02% (SD = 6.49).   Peak detection of the N1 was set between 100-190 ms and the peak of the P200 between 190-280 ms. Furthermore, difference waves were calculated to examine the magnitude difference between the late-potentiation, early-potentiation and pre-potentiation conditions. Peak detection of these difference waves was extracted as well. Scalp electrodes chosen for the analysis were P7, P3, Pz, P4, P8, POz, O1, Oz, and O2.

*Statistical analyses*

Mismatch negativity

To test whether the roving auditory oddball task elicited a significant difference in amplitude between the deviant (tone 1) and standard tone (tone 5), data entered LMMs for both the N170 and the P3a amplitude and latency separately. Tone condition (2 levels) was added as an additional factor to the model. When there was a significant main effect of Tone, separate comparisons between the deviant and standard tone were made per treatment condition per test day to examine whether the difference was consistent during all measurements.

To examine whether the difference in amplitude between the standard and deviant tone differed between treatment conditions and test days, difference waves were calculated (deviant minus standard) for N170 and the P3a, and analyzed with LMMs.

To examine whether there was a repetition suppression effect, LMMs of the baseline N170 and P3a amplitudes were conducted with Tone (5 levels) as an additional factor to check for repetition suppression effect differences between treatment conditions. When there was a significant Electrode by Tone interaction effect, pairwise comparisons were conducted to examine whether all electrodes included in the model showed a repetition suppression effect. In case of a significant Electrode by Tone interaction, subsequent LMMs were conducted with the electrodes that showed a repetition suppression effect. In case there was no significant Electrode by Tone interaction, all electrodes in the model were used in the subsequent LMMs. In case of a three-way interaction effect of Treatment by Test day by Tone, follow-up LMMs were conducted for each test day separately, with Tone as a repeated factor. When a significant Treatment by Tone interaction was present, separate pairwise comparisons were made comparing tone 5 with each preceding tone, per treatment condition.

Visual LTP

LMMs were conducted to check for the effect Input specificity (tetanized vs non-tenatized) and Time (pre-potentiation, early-post, and late-post potentiation) of the N1 and the P200 amplitudes. There was no effect of Input specificity, therefore LMMs were conducted with the averaged data over both the tetanized and non-tetanized conditions. In case of a three-way interaction effect of Treatment by Test day by Time, follow-up LMMs were conducted for each test day separately, with Time as a repeated factor. When a significant Treatment by Time interaction was present, separate pairwise comparisons were made comparing the effect of Time, per treatment condition.

To examine whether the difference in magnitude of the Time conditions differed between treatment conditions and test days, difference waves were calculated (late-post minus pre-potentiation, early-post minus pre-potentiation, late-post minus early-post potentiation) of the N1and the P200, and entered the same LMMs as described above.

**Results**

*Resting state oscillatory power*

*Table S1.* Linear Mixed Models on *baseline* resting state EEG power for both eyes open (EO) and eyes closed (EC) conditions.

| **Delta** | **EO** | **EC** |
| --- | --- | --- |
| Treatment x Electrode | *F*_9,45_= 0.68 | *F*_9,45_= 0.76 |
| Treatment | *F*_1,45_= 0.46 | *F*_1,45_= 0.31 |
| Electrode | *F*_9,45_= 50.33** | *F*_9,45_= 30.35** |
| **Theta** |  |  |
| Treatment x Electrode | *F*_9,45_= 1.67 | *F*_9,45_= 0.59 |
| Treatment | *F*_1,45_= 0.26 | *F*_1,45_< 0.01 |
| Electrode | *F*_9,45_= 25.04** | *F*_9,45_= 12.06** |
| **Alpha** |  |  |
| Treatment x Electrode | *F*_9,45_= 1.49 | *F*_9,45_= 0.93 |
| Treatment | *F*_1,45_= 0.40 | *F*_1,45_= 1.33 |
| Electrode | *F*_9,45_= 9.75** | *F*_9,45_= 9.17* |
| **Beta** |  |  |
| Treatment x Electrode | *F*_9,45_= 1.01 | *F*_9,45_= 0.58 |
| Treatment | *F*_1,45_= 0.01 | *F*_1,45_= 0.28 |
| Electrode | *F*_9,45_= 4.49** | *F*_9,45_= 6.46** |
| **Gamma** |  |  |
| Treatment x Electrode | *F*_9,45_= 1.99 | *F*_9,45_= 0.98 |
| Treatment | *F*_1,45_= 1.91 | *F*_1,45_= 1.52 |
| Electrode | *F*_9,45_= 7.23** | *F*_9,45_= 7.41** |

Note: EO, eyes open; EC, eyes closed. **statistically significant at *p*<.01.

*Table S2.* Linear Mixed Models on *treatment* resting state EEG for both eyes open (EO) and eyes closed (EC) conditions. All interactions with the factor Electrode were non-significant, indicating that effects on power affected the whole default mode network.

| **Treatment by Test day** | **EO** | **EC** |
| --- | --- | --- |
| Delta | *F*_2,438.49_ = 23.86** | *F*_2,435.80_ = 31.82** |
| Theta | *F*_2,438.91_ = 42.92** | *F*_2,436.01_ = 56.19** |
| Alpha | *F*_2,441.53_ = 33.43** | *F*_2,440.40_ = 7.66** |
| Beta | *F*_2,439.10_ = 15.98** | *F*_2,440.21_ = 1.78 |
| Gamma | *F*_2,442.68_ = 0.54 | *F*_2,452.23_ = 6.68** |
| **Treatment** | **EO** | **EC** |
| Delta | *F*_1,451.31_ = 58.35** | *F*_1,451.99_ = 52.55** |
| Theta | *F*_1,446.50_ = 9.64** | *F*_1,451.03_ = 6.16* |
| Alpha | *F*_1,452.78_ = 1.94 | *F*_1,450.95_ = 6.58* |
| Beta | *F*_1,451.35_ = 0.14 | *F*_1,451.23_ = 1.83 |
| Gamma | *F*_1,453.37_ = 5.75* | *F*_1,454.46_ = 2.50 |
| **Test day** | **EO** | **EC** |
| Delta | *F*_2,438.49_ = 29.68** | *F*_2,435.80_ = 69.96** |
| Theta | *F*_2,438.91_ = 43.21** | *F*_2,436.01_ = 42.73** |
| Alpha | *F*_2,441.53_ = 40.65** | *F*_2,440.40_ = 5.25** |
| Beta | *F*_2,439.10_ = 18.29** | *F*_2,440.21_ = 61.73** |
| Gamma | *F*_2,442.68_ = 22.28** | *F*_2,452.23_ = 37.28** |
| **Electrode** | **EO** | **EC** |
| Delta | *F*_9,451.31_ = 7.73** | *F*_9,451.99_ = 6.05** |
| Theta | *F*_9,446.50_ = 3.56** | *F*_9,451.03_ = 1.64 |
| Alpha | *F*_9,452.78_ =2.79** | *F*_9,450.95_ = 3.17** |
| Beta | *F*_9,451.35_ = 0.61 | *F*_9,451.23_ = 0.44 |
| Gamma | *F*_9,453.37_ = 2.52** | *F*_9,454.46_ = 0.82 |

*statistically significant at *p*≤ .05, **statistically significant at *p*≤.01.

**
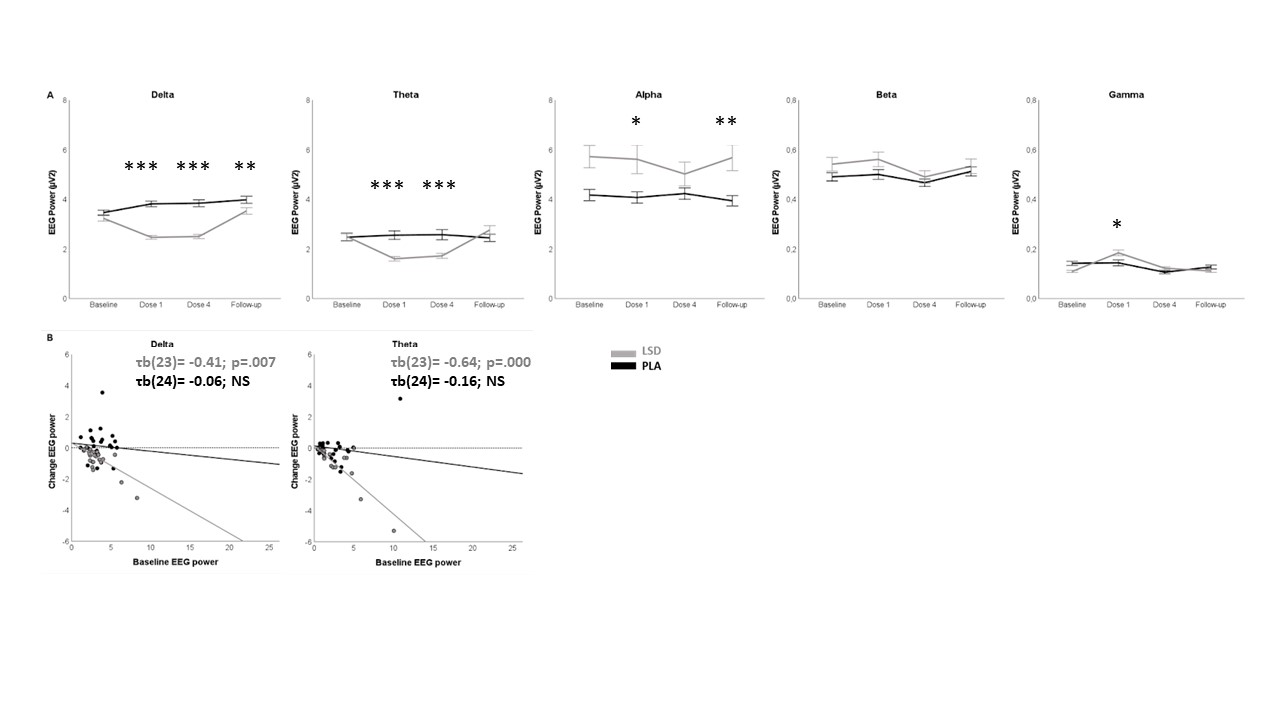
**

*Figure S1.* (A) Mean (SE) resting state EEG power for the LSD (grey) and placebo (black) conditions per test day. (B) Scatterplots of mean change in resting state delta and theta EEG power (the mean over the two dosing days minus baseline, for the LSD (grey) and placebo (black) condition as a function of baseline resting state EEG power. Eyes closed.***= *p*<.001, ** =  *p*<.01, *-p<.05.

*Mismatch negativity*

Paradigm check
Overall, LLMs revealed a main effect of Tone condition for both N170 and the P3a peak voltages (*F*_1,747.55_ =849.62, *p*< .01; *F*_1,763.22_ =924.38, *p*< .01), respectively. Indicating a significantly higher amplitude for the deviant tone (tone 1) compared to the standard tone (tone 5), meaning a successful implementation of the paradigm. Planned pairwise comparisons showed that the difference in amplitude between the deviant and standard tone was significant in both treatment conditions across all test days (all *p’s* < .01). In addition, there was a significant Electrode by Tone interaction effect for both N170 and P3a amplitude (*F*_8,777.34_ =14.51, *p*< .01; *F*_8,753.07_ =13.92, *p*< .01), pairwise comparisons showed that all electrodes included in the model have this significant difference between the standard and deviant tone (all *p* <0.05). LMMs on the difference waves (tone 1 - tone 5) of the MMN and the P3a at baseline, revealed a significant Treatment by Electrode interaction effect on the MMN latency (*F*_8,45_= 1.34, *p*<.01), indicating that the MMN appeared significantly earlier in the LSD condition compared to the placebo condition, this was only shown at the F3 electrode.

Independent samples t-test showed no significant difference between treatment conditions (*t*(42) = 1.42, *p* = .16) in reaction time to the visual distractor on the baseline test day (LSD: *M* = 560.77, *SE* = 26.40; PLA: *M* = 627.00, *SE* = 26.40). Subsequent LMMs including dose 1, dose 4, and follow-up sessions, revealed no significant main effect of Treatment (*F*_1,45.31_= 1.21), Test day (*F*_2,36.31_= 2.47), and Treatment by Test day (*F*_2,36.31_= .10) interaction, on reaction time, indicating that participants in both treatment conditions were equally distracted by the visual task on all test days.

*Table S3.* Linear Mixed Models on the difference waves (tone 1 - tone 5) of the MMN and the P3a at baseline.

|  | **Voltage** | **Latency** |
| --- | --- | --- |
| **MMN** |  |  |
| Treatment x Electrode | *F*_8,45_= 1.77 | *F*_8,45_= 4.08** |
| Treatment | *F*_1,45_= 1.65 | *F*_1,45_= 0.61 |
| Electrode | *F*_8,45_= 16.79** | *F*_8,45_= 1.34 |
| **P3a** |  |  |
| Treatment x Electrode | *F*_8,45_= 0.85 | *F*_8,45_= 0.31 |
| Treatment | *F*_1,45_= 0.15 | *F*_1,45_= 3.50 |
| Electrode | *F*_8,45_= 9.79** | *F*_8,45_= 0.95 |

**statistically significant at *p*≤ 0.01.

*Table S4.* Linear Mixed Models on the MMN difference waves (tone 1- tone 5) after treatment.

| **MMN** | **Voltage** | **Latency** |
| --- | --- | --- |
| Treatment x Test day | *F*_2,381.67_ = 7.05** | *F*_2,394.10_ = 0.65 |
| Treatment x Electrode | *F*_8,406.94_ = 0.56 | *F*_8,408.05_ = 0.35 |
| Test day x Electrode | *F*_16,381.36_ = 0.45 | *F*_16,393.74_ = 0.59 |
| Treatment | *F*_1,397.85_ = 0.29 | *F*_1,407.58_ = 35.90** |
| Test day | *F*_2,381.67_ = 6.80** | *F*_2,394.10_ = 2.75 |
| Electrode | *F*_8,397.82_ = 19.99** | *F*_8,407.52_ = 0.22 |
| **P3a** | **Voltage** | **Latency** |
| Treatment x Test day | *F*_2,373.75_ = 0.80 | *F*_2,388.68_ = 1.22 |
| Treatment x Electrode | *F*_8,388.72_ = 0.62 | *F*_8,399.99_ = 0.33 |
| Test day x Electrode | *F*_16,373.40_ = 0.29 | *F*_16,388.30_ = 0.32 |
| Treatment | *F*_1,387.19_ = 21.63** | *F*_1,401.91_ = 26.91** |
| Testday | *F*_2,373.75_ = 8.65** | *F*_2,388.68_ = 2.37 |
| Electrode | *F*_8,387.17_ = 15.43** | *F*_8,401.85_ = 0.74 |

*statistically significant at *p*≤ 0.05, **statistically significant at *p*≤ 0.01.

*Table S5.* Estimated means (SE) of the MMN difference waves (tone 1 - tone 5) for each test day and treatment condition separately.

|  | **Mean voltage (SE)** | | **Mean latency (SE) in ms** | |
| --- | --- | --- | --- | --- |
| **MMN** | **LSD** | **PLA** | **LSD** | **PLA** |
| **Baseline** | -3.03 (0.23) | -2.62 (0.23) | 176.77 (3.44) | 180.54 (3.37) |
| **Dose 1** | -2.82 (0.11) | -2.79 (0.01) | 177.41 (1.47) | 187.55 (1.44) |
| **Dose 4** | -2.75 (0.10) | -2.56 (0.09) | 175.77 (1.56) | 183.03 (1.52) |
| **Follow-up** | -2.75 (0.11) | -3.15 (0.10) | 176.49 (1.68) | 186.05 (1.61) |
| **P3a** |  | |  | |
| **Baseline** | 2.54 (0.31) | 2.37 (0.30) | 295.81 (3.26) | 304.35 (3.19) |
| **Dose 1** | 2.97 (0.11) | 2.31 (0.11) | 292.79 (1.47) | 298.33 (1.44) |
| **Dose 4** | 3.15 (0.12) | 2.68 (0.12) | 291.77 (1.67) | 301.77 (1.63) |
| **Follow-up** | 3.28 (0.11) | 2.64 (0.11) | 294.51 (1.67) | 302.85 (1.60) |

*
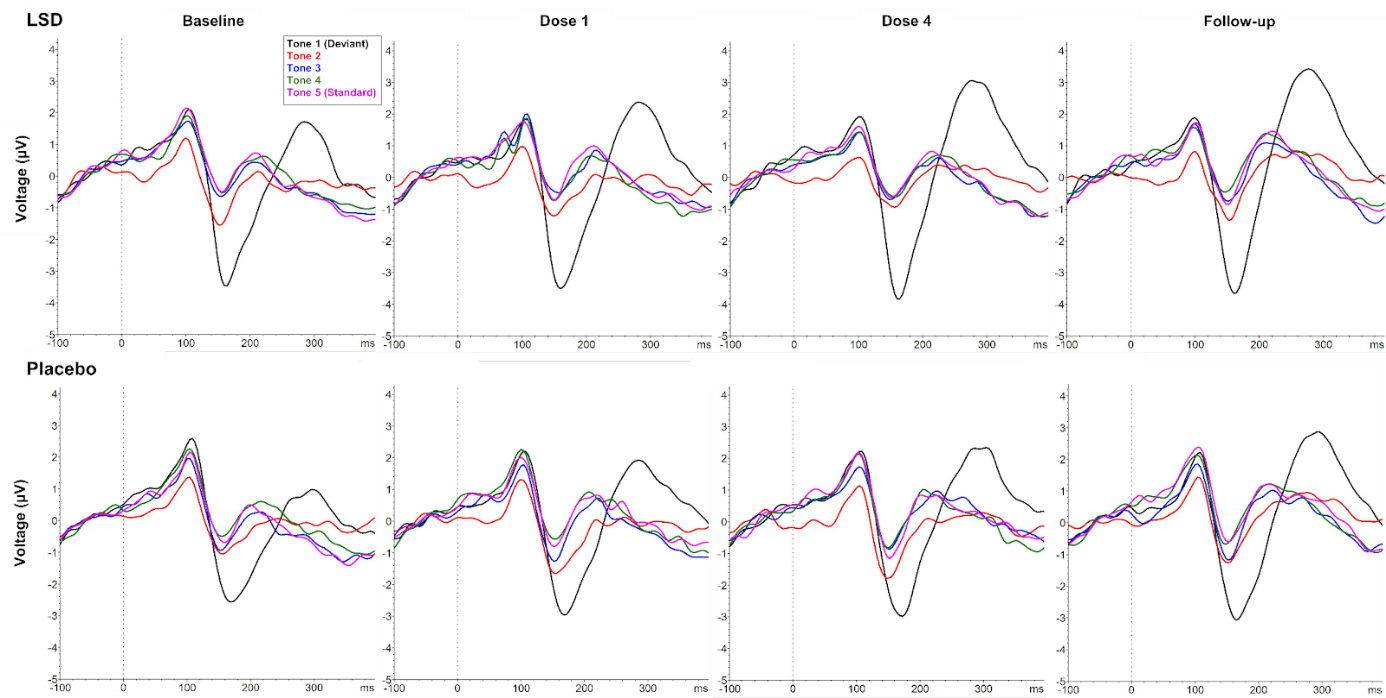
*

*Figure S2.*  ERPs of the roving auditory oddball task per treatment condition (LSD and placebo), on baseline, dose 1, dose 4, and follow-up test day depicted at electrode level of the F4 electrode.

Repetition suppression effect

LMMs on the baseline test day on the N170 amplitude revealed a significant Electrode by Tone interaction effect (*F*_32,1506.97_ = 2.02, *p*< .01). Pairwise comparisons showed that the amplitude of tone 2 was significantly more negative compared to tone 5 (standard) (all *p*<0.05), only on the Fz and F4 electrodes. The other electrodes did only show a significant difference between tone 1 (deviant) and tone 5 (standard), indicating no repetition suppression effect occurred at the other electrodes. Therefore, the next LMMs of the N170 only included the two frontal electrodes.

LMM on the baseline test day on N170 amplitude revealed a significant Tone by Treatment interaction effect (*F*_4,339.86_ = 4.12, *p*< .01). Pairwise comparisons showed that in the LSD condition, habituation took place after 2 repetitions, while this occurred after 1 repetition in the placebo condition. Figure S2 shows the ERPs for each tone, per test day and treatment.

LMMs on the baseline test day on the P3a amplitude revealed no significant Electrode by Tone interaction effect, therefore all 9 electrodes included in the model remained in the model. LMMs on the P200 revealed a Treatment by Tone (*F*_4,1522.47_ = 2.41, *p*< 0.05) interaction and main Tone (*F*_4,1522.47_ = 99.13, *p*< 0.01) effect. Pairwise comparisons showed that in both treatment conditions habituation took place after 1 repetition.

LMMs revealed a significant Treatment by Test day by Tone interaction effect for both the amplitudes of the N170 and the P3a (*F*_8,417.07_ = 2.42, *p*< .05; *F*_8,1831.10_ = 6.52, *p*< .01) respectively. Subsequent LMMs per test day are shown in Table S7.

On dosing session 1, the amplitude of the N170 habituated after 2 repetitions in the LSD group, while this occurred after 1 repetition in the placebo group. For both, dose 4 and the follow-up test day, in both conditions habituation took place after 1 repetition.

As for the amplitude of the P3a, both conditions showed habituation after 1 repetition on dosing session 1, and for the placebo condition, this was also present at dose 4 and the follow-up test day, indicating no repetition suppression effect was present. However, on dose 4 and the follow-up test day, in the LSD condition, 2 repetitions were needed to habituate the P3a amplitude.

*Table S6.* LMMs for each test day separately.

| **N170** | **Dose 1** | **Dose 4** | **Follow-up** |
| --- | --- | --- | --- |
| Treatment x Tone | *F*_4,312.94_= 1.95 | *F*_4,305.43_= 7.97** | *F*_4,311.17_= 2.26 |
| Treatment | *F*_1,85.31_= 0.17 | *F*_1,84.06_= 0.16 | *F*_1,87.53_= 0.12 |
| Tone | *F*_4,394_= 47.79** | *F*_4,305.43_= 42.26** | *F*_4,311.17_= 50.26** |
| Electrode | *F*_1,85.31_< 0.01 | *F*_1,84.06_< 0.01 | *F*_1,87.53_< 0.01 |
| **P3a** | **Dose 1** | **Dose 4** | **Follow-up** |
| Treatment x Tone | *F*_4,1348.69_= 7.38** | *F*_4,1365.06_= 12.48** | *F*_4,1353.34_= 9.33** |
| Treatment | *F*_1,419.29_= 1.72 | *F*_1,420.79_= 8.76** | *F*_1,407.20_= 3.59 |
| Tone | *F*_4,1348.69_= 185.19** | *F*_4,1365.06_= 218.88** | *F*_4,1353.34_= 204.07** |
| Electrode | *F*_8,419.29_= 1.12 | *F*_8,420.79_= 1.96 | *F*_8,407.20_= 3.95** |

**statistically significant at *p*≤ 0.01.

*Visual long-term potentiation*

Input specificity

The LMM showed a main effect of Time (*F_2_*_,2229.95_= 3.58, *p*= 0.03), but no main effect of Input specificity (*F_1_*_,2229.42_= 0.10, *p*= 0.75) or Input specificity by Time interaction (*F_2_*_,2226.72_= 0.46, *p*= 0.63) for the N1amplitude. Pairwise comparisons showed that the amplitude of the N170 was more negative in the late-potentiation than the pre-potentiation condition (95% CI [-0.54, -0.07], *p*= 0.01), other comparisons were non-significant. For the P200 amplitude, the LMM showed a main effect of Time (*F_2_*_,2214.92_= 50.03, *p*< 0.01), but no main effect of Input specificity (*F_1_*_,2213.97_= 0.07, *p*= 0.80) or Input specificity by Time interaction (*F_2_*_,2210.45_= 0.71, *p*= 0.49). Follow-up pairwise comparisons showed that the late-potentiation condition had a more positive P200 amplitude than the pre-potentiation condition and the early-potentiation (95% CI [0.70, 1.08], *p*<0.01; 95% CI [0.60, 0.98], *p*<0.01) respectively; the other comparison was non-significant. Furthermore, for both the N170 and P200 amplitude, there were no significant electrode interaction effects, indicating that all electrodes included in the model showed the same effects.

N1

LMMs on the baseline test day revealed a main effect of Time on the amplitude and latency of the N1 (Table S8). Bonferroni-corrected pairwise comparisons showed that overall the amplitude of the late- and the early-potentiation conditions were more negative compared to pre-potentiation (95% CI [-0.59, -0.25], *p*<0.01; 95% CI [-0.49, -0.19], *p*<0.01) respectively. In addition, the amplitude of the N170 during the late potentiation appeared earlier than the amplitude of the pre-potentiation (95% CI [-4.55, -1.25], *p*< 0.01). There was no significant Treatment by Time interaction, indicating no significant differences in potentiation for the N1amplitude and latency between the two treatment conditions on the baseline test day (Table S7). Furthermore, the magnitude of potentiation at the N1 (difference waves) did also not differ between Treatment conditions (Table S7).

LLMs revealed a significant Treatment by Test day by Time interaction effect on the N1 amplitude (*F*_2,1046.70_= 4.07, *p* <0.05). Follow-up LMMs per test day showed a significant Treatment by Time interaction effect at dose 4 (*F*_2,314.38_= 9.98, *p* <0.01), but not on the follow-up test day (*F*_2,328.32_= 2.76, *p* =0.07). Bonferroni-corrected pairwise comparisons at dose 4, showed that the amplitude of the N170 in both the late- and early-potentiation conditions was significantly more negative compared to the pre-potentiation for the placebo treatment (95% CI [-0.71, -0.08], *p*<0.05; 95% CI [-0.95, -0.40], *p*<0.01) respectively, suggesting a short-term and long-term potentiation. However, there were no significant differences in amplitude of the N1 for both the late- and early-potentiation conditions compared to the pre-potentiation in the LSD condition at dose 4 (95% CI [-0.62, 0.01], *p*=0.06; 95% CI [-0.31, 0.24], *p*=0.81), indicating no significant potentiation has taken place. On the follow-up test day, there was a main effect of Time (*F*_2,328.32_= 4.94, *p* <0.01), indicating that the amplitude of the late-potentiation was significantly more negative compared to the pre-potentiation amplitude (95% CI [-0.42, -0.08], *p*<0.01). There were no significant effects on the latency of the N170.

LMMs on the amplitude of the difference waves of the N1 revealed a Treatment by Test day interaction effect (Table S9). Pairwise comparisons revealed no significant differences in amplitude between treatment conditions and test days. LLMs revealed a significant Treatment by Test day by Time interaction effect on the latency of the N1 difference wave (*F*_2,1093.30_= 8.79, *p* <0.01). Follow-up LLMs showed a Treatment by Time interaction effect on dose 4 and follow-up (*F*_2,357.54_= 25.46, *p* <0.01; *F*_2,332.59_= 4.11, *p* <0.05), respectively. Pairwise comparisons revealed that the amplitude of the early minus pre-potentiation appeared significantly earlier in the LSD condition compared to the placebo condition during dosing session 4 (95% CI [3.85, 13.27], *p*< 0.01). In addition, the amplitude of the late minus pre-potentation wave appeared significantly later compared to the amplitude of the early minus pre-potentiation wave in the LSD condition at dose 4 (95% CI [5.37, 10.66], *p*< 0.01). Pairwise comparisons between treatments and difference waves on the follow-up test day were not significant.

P200

There was a significant Treatment by Time interaction effect for both the amplitude and latency of the P200 on the baseline test day (Table S8). Bonferroni-corrected pairwise comparisons revealed that both treatment groups showed a significantly more positive P200 amplitude in the late potentiation compared to the pre-potentiation (LSD: 95% CI [0.29, 0.75], *p<* 0.01; Placebo: (95% CI [0.57, 1.02], *p* < 0.01). However, the placebo group also showed a significantly less positive amplitude in the early-potentiation condition compared to pre-potentiation (95% CI [-0.48, -0.11], *p*< 0.01). As for the latency, both treatment conditions showed that P200 amplitude appeared earlier in the late-potentiation than in the pre-potentiation (LSD: 95% CI [-4.83, -0.46], *p*< 0.05; Placebo: 95% CI [-6.41, -2.17], *p* <0.01).

LMMS on the difference waves of the P200 showed a main effect of Time and Time by Treatment interaction effect on the baseline test day (Table S8). Pairwise comparisons revealed that in both treatment conditions, the P200 was more positive for the late-potentiation minus pre-potentiation compared to the early- minus pre-potentiation wave (LSD: 95% CI [0.14, 0.62], *p*< 0.01; Placebo: 95% CI [0.62, 1.09], *p* <0.01). This indicates that the magnitude of the LTP was more positive compared to the magnitude of the early potentiation.

*Table S7.* LMMs of the amplitude and latency of the N1 and P200 peak, and of the difference between time waves on the baseline test day*.*

|  |  | |
| --- | --- | --- |
| **N1** | **Voltage** | **Latency** |
| Treatment x Time | *F*_2,361_= 2.36 | *F*_2,361_= 1.18 |
| Treatment | *F*_1,40.78_= 0.44 | *F*_1,41.16_= 0.14 |
| Time | *F*_2,361_= 14.42** | *F*_2,361_= 6.01** |
| Electrode | *F*_8,319.68_= 2.55* | *F*_8,322.51_= 20.12** |
| **P200** |  |  |
| Treatment x Time | *F*_2,361_= 3.14* | *F*_2,361_= 3.09* |
| Treatment | *F*_1,41.59_= 0.04 | *F*_1,40.48_= 0.85 |
| Time | *F*_2,361_= 45.58** | *F*_2,361_= 15.51** |
| Electrode | *F*_8.248.92_= 46.51** | *F*_2,317.18_= 12.95** |
|  | **Difference waves between time** | |
| **N1** | **Voltage** | **Latency** |
| Treatment x Time | *F*_2,362_= 0.14 | *F*_2,362_= 0.88 |
| Treatment | *F*_1,42.63_= 0.01 | *F*_2,42.63_= 0.51 |
| Time | *F*_2,362_= 1.57 | *F*_2,362_= 0.18 |
| Electrode | *F*_8,346.35_= 2.03* | *F*_2,309.71_= 0.63 |
| **P200** |  |  |
| Treatment x Time | *F*_2,362_= 4.28* | *F*_2,362_= 0.78 |
| Treatment | *F*_1,352.88_= 0.26 | *F*_1,40.93_= 0.20 |
| Time | *F*_2,362_= 27.29** | *F*_2,362_= 6.46** |
| Electrode | *F*_2,359.77_= 0.1.34 | *F*_8,302.81_= 1.26 |

*statistically significant at *p*≤ 0.05, **statistically significant at *p*≤ 0.01.

All interactions with the factor Electrode were non-significant.

LMMs on the P200 revealed a significant Treatment by Test day interaction and a main effect of Time effect on both the amplitude (*F*_1,1016.39_= 14.25, *p* <0.01; *F*_2,1071.46_= 25.91, *p* <0.01) and the latency (*F*_1,1052.35_= 5.38, *p* <0.05; *F*_2,1077.38_= 14.64, *p* <0.01) of the P200 respectively. Follow-up pairwise comparisons between Treatment and Test days revealed that overall the P200 amplitude was significantly less positive at dose 4 in the LSD condition compared to the follow-up test day (95% CI [-0.83, -0.43], *p*<0.01), and the peak appeared later (95% CI [0.43, 3.26], *p* <0.05). Overall, the amplitude of the late potentiation was significantly more positive and appeared earlier compared to pre- and early-potentiation (95% CI [0.70, 1.25], *p*<0.01; 95% CI [0.45, 1.00], *p* <0.01) respectively.

The LMM on the amplitude of the difference waves at the P200, revealed a Treatment by Test day interaction effect, and a main effect of Time (Table S8). Separate pairwise comparisons between treatments and test days revealed that the overall amplitude of the difference waves was significantly less positive in the LSD condition compared to the placebo condition at dose 4 (95% CI [-1.13, -0.33], *p*<0.01), this difference was absent on the follow-up test day (95% CI [-0.49, 0.32], *p*=0.67). In addition, the amplitude in the LSD group was less positive at dose 4 compared to the follow-up (95% CI [-0.40, -0.04], *p*< 0.05). The amplitude in the placebo condition was significantly more positive at dose 4 compared to the follow-up test day (95% CI [0.26, 0.59], *p*<0.01).

LMMs on the latency of the difference waves at the P200 revealed a Treatment by Time interaction effect (*F*_2,1050.54_= 12.03, *p*<0.01). Pairwise comparisons revealed that overall the amplitude of the P200 difference waves appeared later earlier at dose 4 compared to follow-up in the LSD condition (95% CI [-5.71, -2.15], *p*<0.01).

*Table S8.* LMMs of the amplitude and latency of the N1 and P200 peak, and of the difference between time waves after treatment

|  | **Main time waves** | |
| --- | --- | --- |
| **N1** | **Voltage** | **Latency** |
| Treatment x Test day x Time | *F*_2,1046.70_= 4.07* | *F*_2,1081.21_= 0.76 |
| Treatment x Test day | *F*_1,1018.19_= 24.40** | *F*_1,1043.19_= 0.60 |
| Treatment x Time | *F*_2,1077.03_= 0.67 | *F*_2,1079.07_= 2.21 |
| Test day x Time | *F*_2,1046.74_= 3.17* | *F*_2,1081.24_= 1.13 |
| Treatment | *F*_1,40.92_= 0.81 | *F*_1,41.08_= 0.27 |
| Test day | *F*_1,1018.36_= 7.61** | *F*_1,1043.43_= 18.86** |
| Time | *F*_2,1077.20_= 1.21 | *F*_2,1079.32_= 0.70 |
| Electrode | *F*_8,1075.45_= 8.68** | *F*_8,1076.76_= 44.93** |
| **P200** |  |  |
| Treatment x Test day x Time | *F*_2,1048.45_= 1.88 | *F*_2,1097.90_= 1.06 |
| Treatment x Test day | *F*_1,1016.39_= 14.25** | *F*_1,1052.35_= 5.38* |
| Treatment x Time | *F*_2,1071.23_= 0.80 | *F*_2,1077.14_= 0.71 |
| Test day x Time | *F*_21048.89_= 0.07 | *F*_2,1097.93_= 1.08 |
| Treatment | *F*_1,41.01_= 0.01 | *F*_1,40.88_= 0.54 |
| Test day | *F*_1,1016.58_= 29.42** | *F*_1,1052.62_= 2.09 |
| Time | *F*_2,1071.46_= 25.91** | *F*_2,1077.38_= 14.64** |
| Electrode | *F*_8.1069.24_= 172.28** | *F*_8,1074.03_= 25.97** |
|  | **Difference waves between time** | |
| **N1** | **Voltage** | **Latency** |
| Treatment x Test day x Time | *F*_2,1127.58_= 1.83 | *F*_2,1093.30_= 8.79** |
| Treatment x Test day | *F*_1,1095.14_= 4.88* | *F*_1,1050.26_= 3.23 |
| Treatment x Time | *F*_2,1033.51_= 0.04 | *F*_2,1048.04_= 25.69** |
| Test day x Time | *F*_2,1127.53_= 4.59** | *F*_2,1093.30_= 1.50 |
| Treatment | *F*_1,40.59_= 1.66 | *F*_1,40.83_= 0.36 |
| Test day | *F*_1,1095.23_= 0.32 | *F*_1,1050.40_= 13.86** |
| Time | *F*_2,1033.69_= 9.88** | *F*_2,1048.16_= 1.89 |
| Electrode | *F*_8,1023.15_= 7.66** | *F*_8,1042.71_= 0.51 |
| **P200** | **Voltage** | **Latency** |
| Treatment x Test day x Time | *F*_2,1125.30_= 0.70 | *F*_2,1098.80_= 2.40 |
| Treatment x Test day | *F*_1,1084.31_= 27.36** | *F*_1,1053.22_= 3.84 |
| Treatment x Time | *F*_2,1067.99_= 2.93 | *F*_2,1050.54_= 12.03** |
| Test day x Time | *F*_2,1125.29_= 0.39 | *F*_2,362_= 4.28* |
| Treatment | *F*_1,40.64_= 4.58* | *F*_2,1098.80_= 1.10 |
| Test day | *F*_1,1084.44_= 2.83 | *F*_1,1053.35_= 19.70** |
| Time | *F*_2,1068.07_= 76.31** | *F*_2,1050.66_= 2.56 |
| Electrode | *F*_8,1060.88_= 12.93** | *F*_8,1045.02_= 2.88** |

*statistically significant at *p*≤ 0.05, **statistically significant at *p*≤ 0.01.

All interactions with the factor Electrode were non-significant.
